# A first insight into the genomic background of *Ilex pubescens* (Aquifoliaceae) by flow cytometry and genome survey sequencing

**DOI:** 10.1186/s12864-023-09359-5

**Published:** 2023-05-19

**Authors:** Peng Zhou, Qiang Zhang, Jiao Li, Fei Li, Jing Huang, Min Zhang

**Affiliations:** 1 Jiangsu Academy of Forestry, 109 Danyang Road, Dongshanqiao, Nanjing, 211153 China; 2 Co-Innovation Center for Sustainable Forestry in Southern China, Key Laboratory of State Forestry and Grassland Administration On Subtropical Forest Biodiversity Conservation, College of Biology and the Environment, Nanjing Forestry University, Nanjing, 210037 China

**Keywords:** Genome survey, Next-generation sequencing, Flow cytometry, Microsatellite, *Ilex pubescens*

## Abstract

**Background:**

*Ilex pubescens* is an important traditional Chinese medicinal plant with many naturally occurring compounds and multiple pharmacological effects. However, the lack of reference genomic information has led to tardiness in molecular biology research and breeding programs of this plant.

**Results:**

To obtain knowledge on the genomic information of *I. pubescens,* a genome survey was performed for the first time by next generation sequencing (NGS) together with genome size estimation using flow cytometry. The whole genome survey of *I. pubescens* generated 46.472 Gb of sequence data with approximately 82.2 × coverage. K-mer analysis indicated that *I. pubescens* has a small genome of approximately 553 Mb with 1.93% heterozygosity rate and 39.1% repeat rate. Meanwhile, the genome size was estimated to be 722 Mb using flow cytometry, which was possibly more precise for assessment of genome size than k-mer analysis. A total of 45.842 Gb clean reads were assembled into 808,938 scaffolds with a relatively short N50 of 760 bp. The average guanine and cytosine (GC) content was 37.52%. In total, 197,429 microsatellite motifs were detected with a frequency of 2.8 kb, among which mononucleotide motifs were the most abundant (up to 62.47% of the total microsatellite motifs), followed by dinucleotide and trinucleotide motifs.

**Conclusion:**

In summary, the genome of *I. pubescens* is small but complex with a high level of heterozygosity. Even though not successfully applied for estimation of genome size due to its complex genome, the survey sequences will help to design whole genome sequencing strategies and provide genetic information support for resource protection, genetic diversity analysis, genetic improvement and artificial breeding of *I. pubescens*.

**Supplementary Information:**

The online version contains supplementary material available at 10.1186/s12864-023-09359-5.

## Background

*Ilex pubescens* Hook. et Arn. (2n = 40), also known as ‘Mao-dong-qing’ in Chinese, is an evergreen shrub belonging to the genus *Ilex* in the family Aquifoliaceae [1–3]. This species is distributed in the wild in southern China with a natural distribution limited to an altitude of 100–1000 m, which is particularly prevalent in Guangdong and Guangxi [3]. The roots of *I. pubescens* are commonly used as Chinese herbal medicine for the treatment of cardiovascular disease, cerebral thrombosis and hypercholesterolemia [4–6]. The therapeutic effects are attributed to the bioactivities of the naturally occurring compounds in this herb. More than 200 compounds have been isolated and identified from this plant, among which a substantial proportion were reported to be triterpenes [7, 8]. However, the molecular mechanisms of the biosynthesis of these medicinal active ingredients remains unclear.

With the increasing demand for herbal drugs and natural health products, studies focusing on *I. pubescens* have attracted widespread attention in recent years [7, 9]. As a valuable wild and medicinal plant resource in China, the supply of *I. pubescens* for medicinal materials mostly depended on the exploitation of wild populations [10]. However, with the increasing demand, excessive exploitation and rapid shrinking of habitat caused by human activities, wild populations of *I. pubescens* had decreased in recent years, which was difficult to meet the increasing demand and sustainable use [11]. In addition, because medicinal products of *I. pubescens* have different quality requirements, it is essential to breed varieties with different characteristics through genetic improvement [4]. Therefore, the domestication and cultivation of this species is an effective way [12, 13]. With the development of cultivation techniques, breeding of different varieties will be the focus of *I. pubescens* in future work, while the lack of genetic and genomic data currently had led to limited improvement in its breeding programs*.*

Genome sequencing has been an important step to decipher the genetic structure and accelerate genetic improvements in traits of interest in organisms [14, 15]. Exploring the genes related to the effective components and excellent agronomic characters of plants, and analyzing the metabolic pathway and regulatory mechanism from the genome-wide level can lay the foundation for the improvement of medicinal plant varieties and the protection of genetic resources [16–18]. Whereas, due to the highly complex genetic background of some plant species, particularly woody plants and medicinal plants [18–21], genome survey sequencing is a very necessary and important step before large-scale genome sequencing of these kinds of species, which not only gives a preliminary understanding of the genomic characteristics, but also can generate a large amount of genetic information and molecular markers for plant breeding through high-throughput next-generation sequencing (NGS) [14, 22–24].

In this study, flow cytometry and genome survey sequencing were adopted to explore the genome size and characteristics of *I. pubescens*. The acquisition of genomic information in this study would enhance the understanding of genome and provide a reference for subsequent genome-wide sequencing and further molecular elucidation of the synthesis of the medicinal active ingredients in *I. pubescens*. In the meantime, the molecular markers developed based on genome survey would be helpful to genetic diversity evaluation and could accelerate the progress of genetic improvement, artificial breeding and culturing of *I. pubescens*.

## Results

### Genome size estimation by flow cytometry

**Fig. 1:**
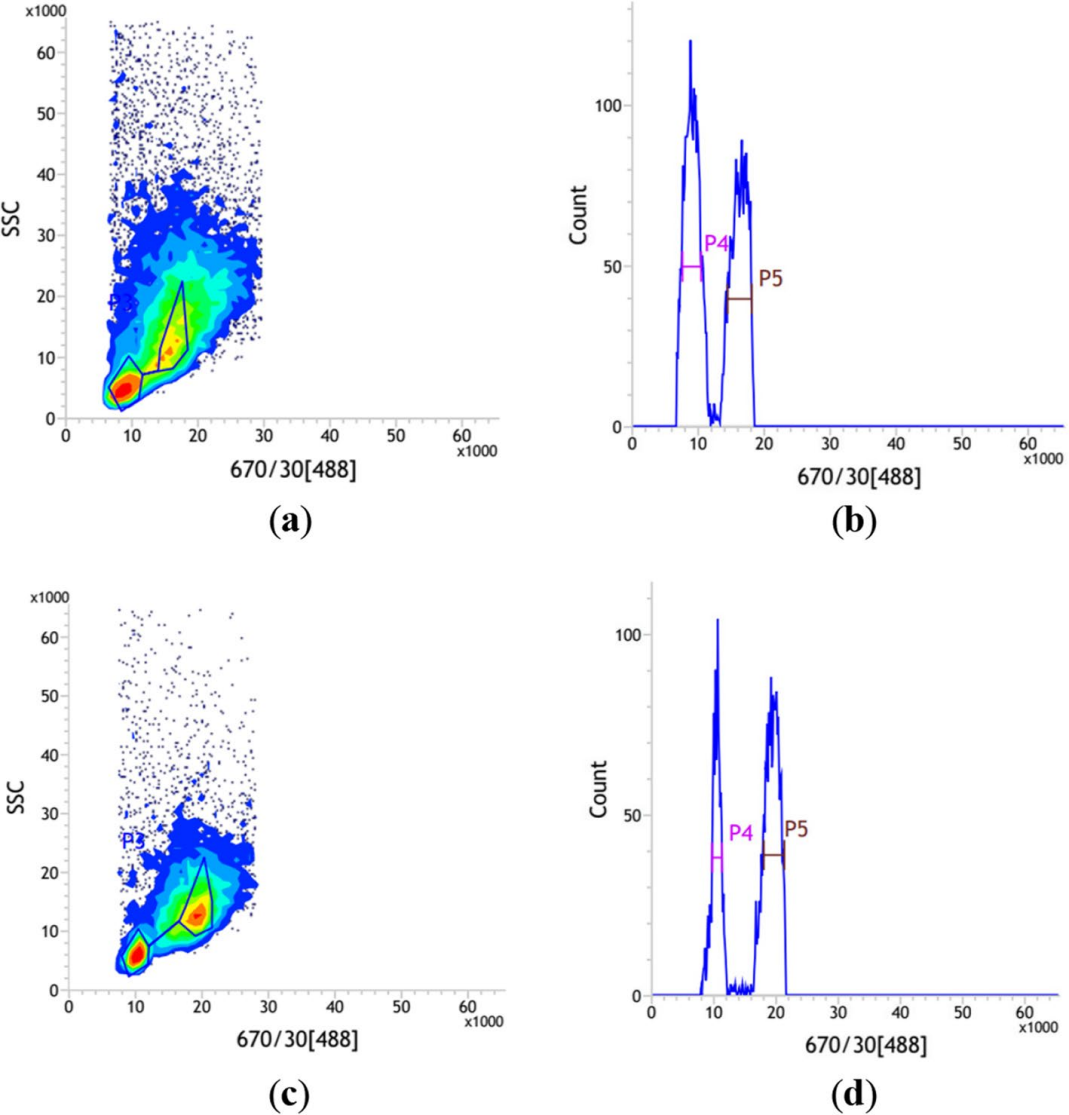
The result of FCM analysis for *I. pubescens* samples simultaneously processed with rice. **a**, **b** The representative female sample; **c**, **d** The representative male sample. (**a**, **c**) Scatterplot on side scatter (SSC) versus PI fluorescence with manually drawn polygon gate; (**b**, **d**) Histogram of relative fluorescence intensity derived from nuclei isolated from rice and *I. pubescens* processed simultaneously. Peak 4 represents G_0_/G_1_ nuclei of rice, peak 5 represents G_0_/G_1_ nuclei of *I. pubescens* sample

The flow cytometric analysis yielded high-resolution histogram (Fig. 1) with the mean CV of 4.81% and 3.68% for the internal standard rice and *I. pubescens*, respectively (Table 1). The results showed that the average genome size of diploid *I. pubescens* was estimated to be approximately 722 ± 8 Mb. The genome size of males (729 ± 13 Mb) was estimated to be larger than that of females (715 ± 7 Mb). However, the difference between male and female samples in genome size detected using flow cytometry was not significant (*P* > 0.05).

**Table 1:** Statistics of flow cytometry data

Sex	Genome size (Mb)	CV (%) of standard	CV (%) of samples
Female	715 ± 7	4.65	2.89
Male	729 ± 13	4.98	3.78
**Average**	722 ± 8	4.81	3.68

*Abbreviations*: *CV* the coefficient variation value of peak

### Genome survey sequencing and quality evaluation

**Fig. 2:**
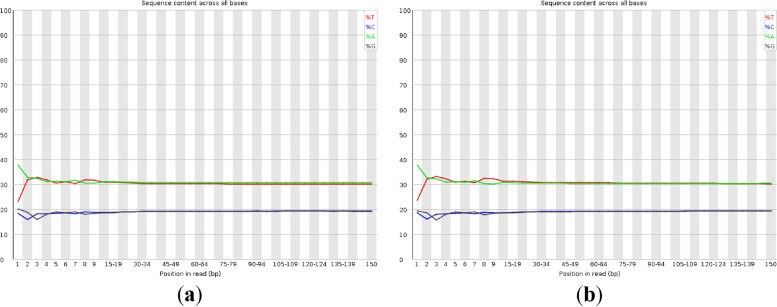
Distribution figure of GC content. **a** The GC content distribution in read-1. **b** The GC content distribution in read-2

The libraries of paired-end sequencing with 350 bp short-inserts of *I. pubescens* were constructed. A total of 46.472 Gb of raw bases, which was an approximately 82.2-fold coverage of the estimated genome size, were generated by the DNBSEQ-T7 sequencing platform. After filtering and correction, a total of 45.842 Gb of clean bases were obtained with the Q20 and Q30 values of 97.74% and 92.605%, respectively (Table 2), indicating that the high-throughput sequencing was highly accurate. In addition, the guanine plus cytosine (GC) content of the raw reads was 38.55%. The proportion of single bases is usually used to determine whether AT and GC separation are present separately. As show in Fig. 2, the proportion of A, G, C and T were close with no obvious GC bias. All the results demonstrated that the sequencing quality was good.

**Table 2:** Statistics of sequencing data and quality assessment of *I. pubescens*

Number of raw reads	Raw base (Gbp)	Clean base (Gbp)	Q20 (%)	Q30 (%)	GC content (%)	Average Quality
309,811,880	46.472	45.842	97.74	92.605	38.55	35.74

*Abbreviations*: *Q20* percentage of bases with quality value ≥ 20, *Q30* percentage of bases with quality value ≥ 30

### Genomic characteristics predicted by k-mer analysis

**Fig. 3:**
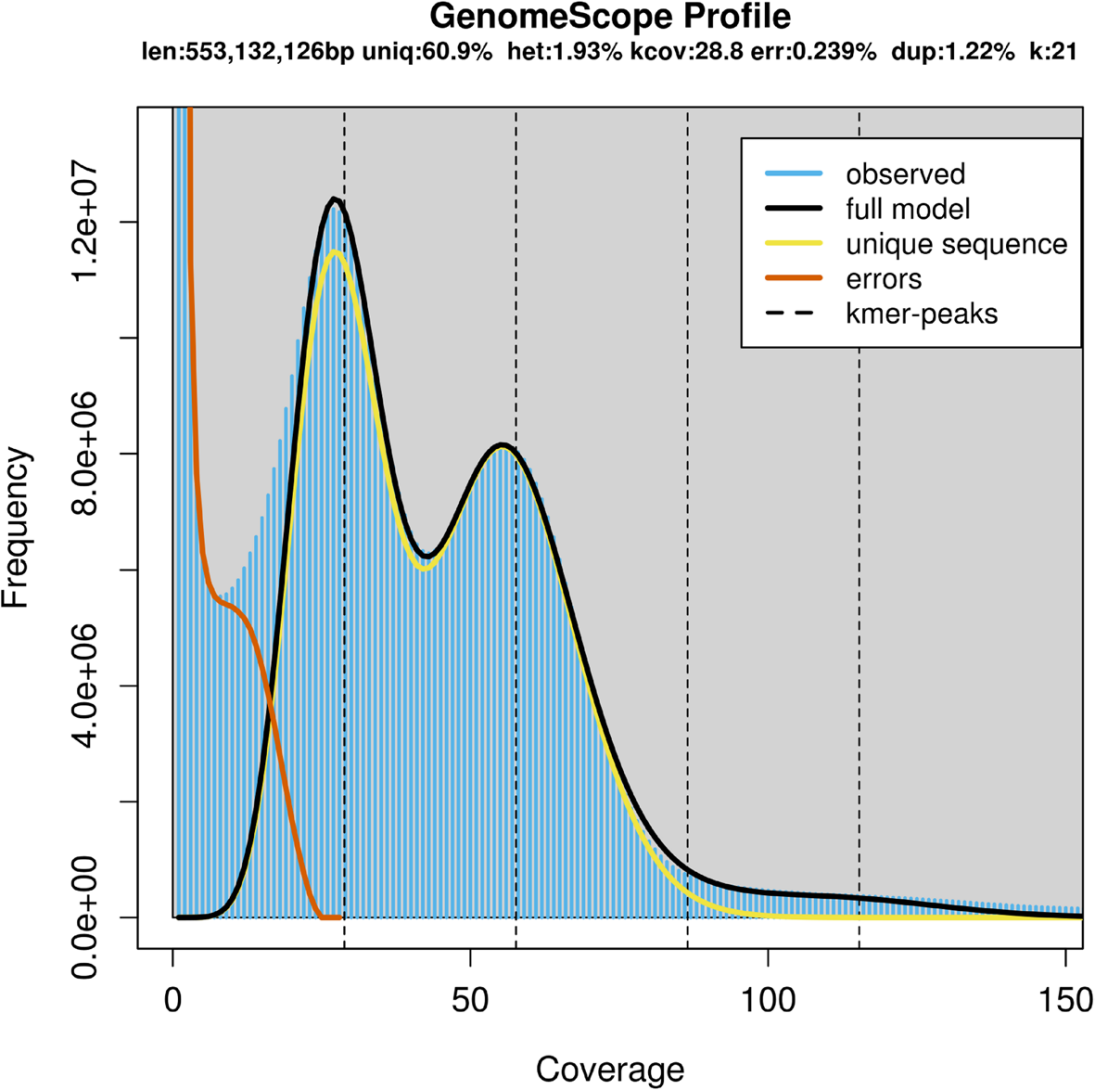
K-mer (k = 21) distribution calculated by Genomescope. Blue bars represent the observed k-mer distribution; black line represents the modelled distribution without the k-mer errors (red line) and up to a maximum k-mer coverage specified in the model (yellow line). len, estimated genome length; uniq, unique portion of the genome (nonrepetitive elements); het, genome heterozygosity; err, the sequencing error rate

The entire clean reads were used to predict the genomic characteristics of *I. pubescens* by k-mer analysis. Based on the 21-mer frequency distribution, the genome size was estimated to be 553 Mb, which was 77% of the size (722 Mb) estimated using flow cytometry, and the heterozygosity rate and repeat rate were 1.93% and 39.1%, respectively (Fig. 3). Therefore, the genome of this species belongs to the complex genome with high heterozygosity. Additionally, the sequencing error rate was 0.239%.

### Preliminary genome assembly

**Fig. 4:**
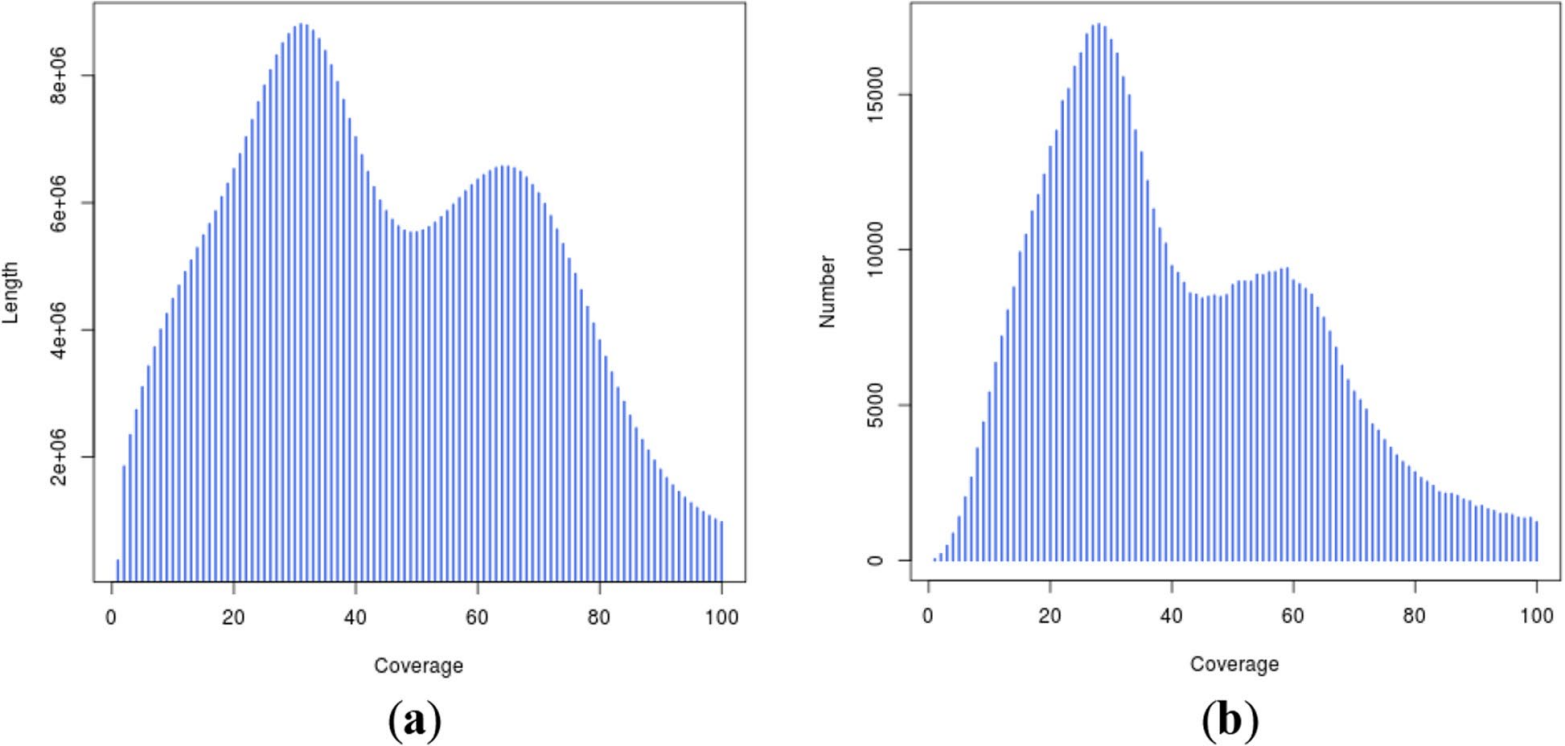
Distribution figure of scaffold. **a** Coverage depth and length. The x-axis represents the coverage and the y-axis is the sequence length. **b** Coverage depth and number. The x-axis represents the coverage and the y-axis is the sequence number

All the high quality data were assembled *de novo* (k-mer = 21) by using the *de Bruijn* graph-based SOAPdenovo software. A total of 8,584,072 raw contigs were obtained with the contig N50 of 211 bp, and the total length of raw contigs was 1,455,860,256 bp (Table 3). Scaffolds larger than 300 bp were selected to avoid low-quality sequences. The assembled *I. pubescens* genome was consisted of 808,938 scaffolds with a total length of 564,024,385 bp and the scaffold N50 of 760 bp (Table 3). According to the significant peaks of the scaffold distribution (Fig. 4), the peak at approximately 57 × was a homozygous peak, and the peak located halfway in front of the homozygous peak was the heterozygous peak, which also proved the existence of high heterozygosity in the *I. pubescens* genome.

**Table 3:** Statistics of assembled genome sequences of *I. pubescens*

	**Total length (bp)**	**Total number**	**Max length (bp)**	**Min length (bp)**	**N20 length (bp)**	**N50 length (bp)**	**N80 length (bp)**
Contig	1,455,860,256	8,584,072	33,238	64	728	211	95
Scaffold	564,024,385	808,938	33,238	300	2,488	760	406

### Guanine plus cytosine (GC) content and distribution status

**Fig. 5:**
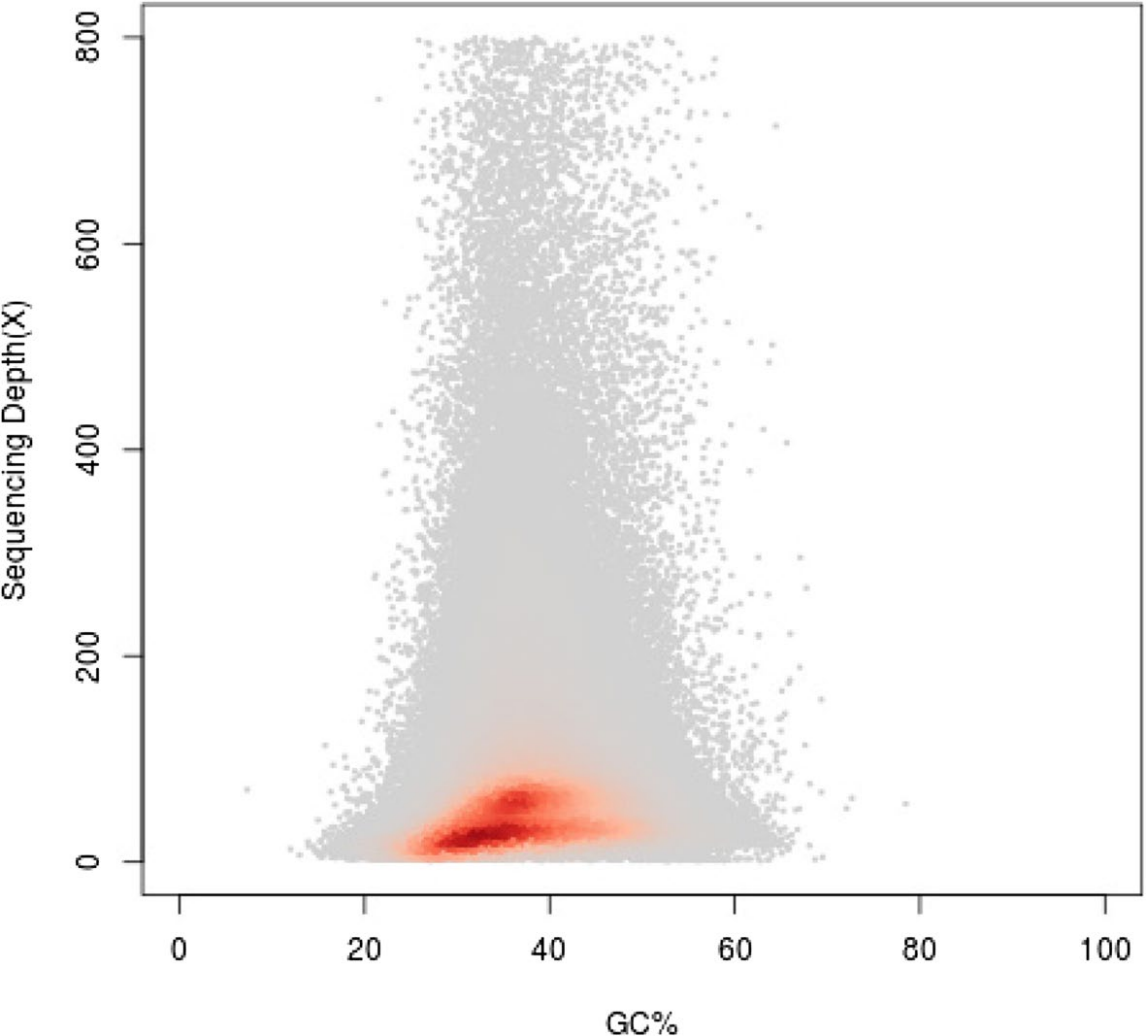
Guanine plus cytosine (GC) content and depth correlation analysis. The x-axis represents the GC content and the y-axis is the sequence depth

According to the GC-Depth scatterplot graph built by scaffolds larger than 300 bp, it could be judged whether there was obvious GC bias in the sequencing results, and also determined whether there was bacterial contamination. As shown in Fig. 5, the GC content of the windows was mostly concentrated in the range of 20–50%, with the average GC content of the scaffolds was 37.52% after calculation. There was no apparent abnormal accumulation area, suggesting that the DNA sample for sequencing was not polluted by DNA from other species. In addition, the GC depth distribution was divided into two layers (Fig. 5), mainly due to the high heterozygosity [17, 25, 26].

### Identification and characteristics of microsatellite motifs

**Fig. 6:**
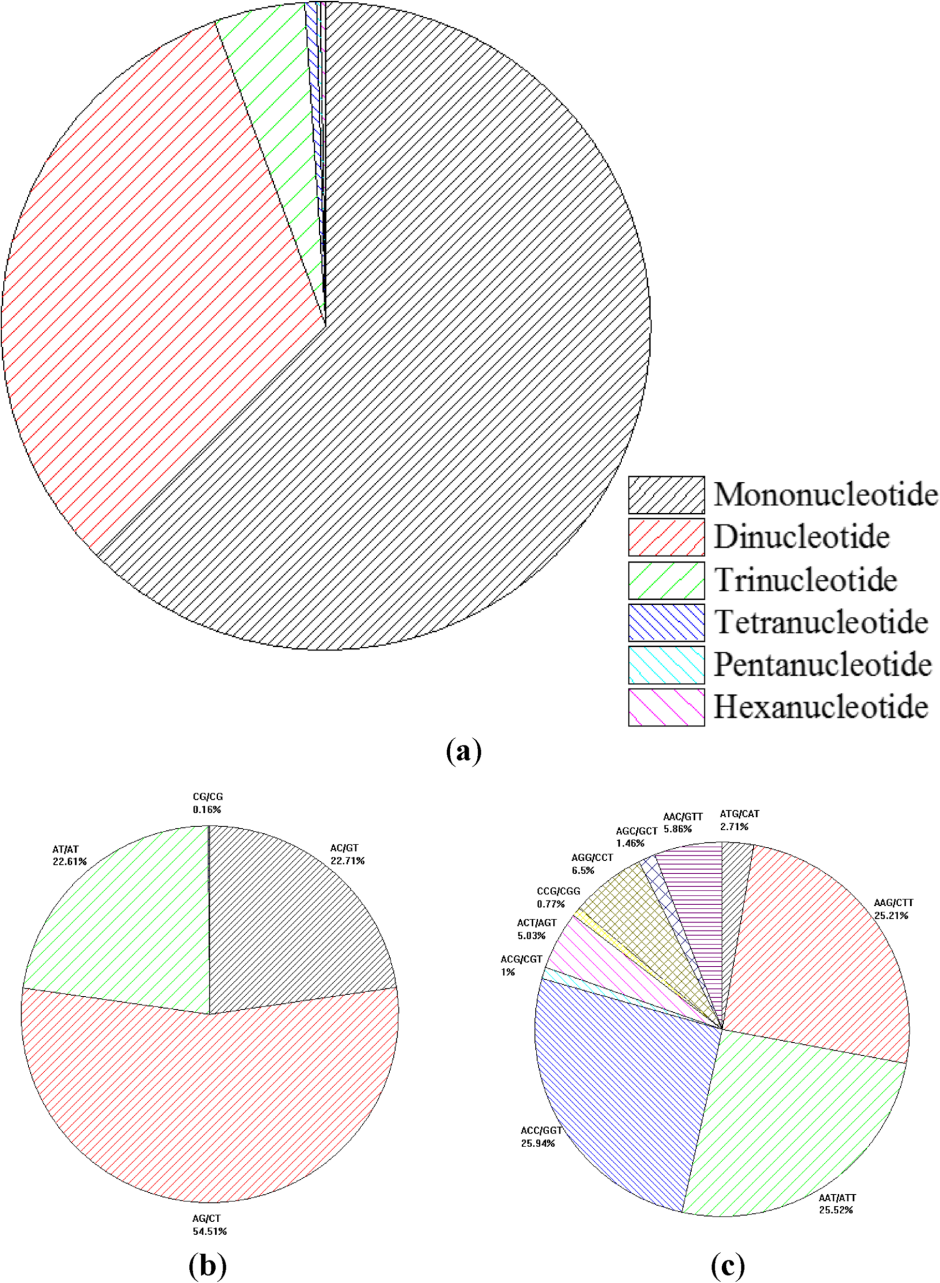
Characteristics of microsatellite motifs. **a** Frequency of different microsatellite motifs. **b** Frequency of different dinucleotide motifs. **c** Frequency of different trinucleotide motifs

A total of 197,429 microsatellite motifs were identified from the genome assembly of *I. pubescens* (Additional file 1: Table S1). Among them, the mononucleotide motifs were the most prevalent, accounting for 62.47% (123,333) of the total microsatellite motifs, followed by dinucleotides (63,069; 31.95%), trinucleotides (8,971; 4.54%), tetranucleotides (1,128; 0.57%), pentanucleotides (388; 0.20%) and hexanucleotides (540; 0.27%) (Fig. 6a). Among the mononucleotide repeats, A/T repeats were the predominant type, accounting for 92.56% of the total repeat units. In the dinucleotides, the most frequent motif was AG/CT (54.51%), followed by AC/GT (22.71%), AT/AT (22.61%) and CG/CG (0.16%) (Fig. 6b). In the trinucleotides, the predominant motifs ACC/GGT, AAT/ATT and AAG/CTT accounted for 25.94%, 25.52% and 25.21%, respectively (Fig. 6c).

## Discussion

### Genome size prediction via flow cytometric analysis

Genome size is an important attributes of the genome of an organism, and accurate estimation of genome size is pre-requisite before the genome sequencing [21]. Flow cytometry has become a well-recognized method for the prediction of genome size before plant genome sequencing [27, 28]. Until now, the published genome size obtained by sequencing of Aquifoliaceae plants ranged from 727 to 804 Mb [29–31]. The genome size of *I. pubescens* was estimated to be close to the data obtained from Aquifoliaceae plants previously, indicating that the result of flow cytometry in this study was credible. According to the classification criteria proposed by Soltis et al. [32]*, I. pubescens* has a very small genome size.

### The valuable information for whole genome project provided by the genome characteristics

Heterozygosity and repeat rates of genome are critical factors affecting the quality of genome assembly and subsequent analysis [14, 22, 25]. The genome of *I. pubescens* was considered as lowly repetitive genome [33], but the high heterozygosity indicated the high complexity in the genome of this species, possibly due to the dioecious mating system in *Ilex* genus [34]. Previous studies have suggested that if the genomic heterozygosity exceeded 1%, the genome-scale *de novo* assembly was considered to be quite difficult [22]. The N50 lengths of contigs and scaffolds were relatively short, mainly because of a high heterozygosity of *I. pubescens* genome contributing to the unsatisfactory assembly results, which was similar to those results in other studies [20, 21, 35]. GC content was another important factor contributing to sequencing bias on the Illumina sequencing platform, which exceeding the normal range will result in reduced coverage in sequenced regions and seriously affect genome assembly [15, 36]. In this study, the GC content of *I. pubescens* was medium, which fell within the acceptable range of 25–65% for genome assembly [20, 25, 37, 38]. Therefore, based on the complex characteristics, the genome of *I. pubescens* will be relatively difficult to assemble by traditional approaches and higher-depth third-generation sequencing may yield better assembly results [17, 20, 39].

### The inefficiency of k-mer analysis for estimating genome size for complex genome

In recent years, the k-mer method combined with genome survey sequencing has been successfully applied for the estimation of genome size for many non-model species without prior knowledge [18, 20, 40]. However, the k-mer analysis for estimating the genome size of complex genome was still not powerful, which accuracy of estimated genome size would be decreased [36]. For example, in the study of *Camellia chekiangoleosa*, the genome size estimated using k-mer analysis was 2.33 Gb with 1.75% heterozygosity rate, however, the assembly result of three-generation sequencing was 2.73 Gb [41]. In the present study, the result of genome size estimation by k-mer analysis was aslo far below that of flow cytometry, which might been affected by the existence of high heterozygote in the genome of *I. pubescens* [36, 42]. In short, genome survey could not be successfully applied for estimation of *I. pubescens* genome size.

### Genome-wide microsatellite analysis

The assessment of genetic diversity and structure is one of the major goals of population management and conservation biology, which should ideally be achieved by utilizing polymorphic and informative markers [40]. Microsatellite markers have become one of the most popular molecular markers and one of the most powerful tools for genetic diversity, linkage mapping, germplasm identification and evolution analysis [40, 43, 44]. Therefore, the genome-wide SSR markers being characterized and developed will significantly contribute to the *I. pubescens* genomic resources and facilitate the genetic and genomic studies. The tendency of the motif frequency in the studied species was similar to that in other plant species, with the mononucleotide motifs being the predominant type [17, 18, 20, 21].

## Conclusions

In the present study, the genome size and characteristics of the genome of *I. pubescens* were preliminarily investigated for the first time, which greatly enriched the genomic resources for the further excavation and utilization of this species*.* As the genome of *I. pubescens* is small but complex with high heterozygosity, for future whole-genome sequencing, the second-generation and third-generation sequencing technologies combined with Hi-C and BioNano for supplement are recommended to yield better genome assembly results. The newly identified genome-wide SSR motifs in this study may provide basic molecular markers for genetic and molecular biology studies, key genes analysis for effective components synthesis as well as artificial breeding of *I. pubescens*.

## Methods

### Plant materials

**Fig. 7:**
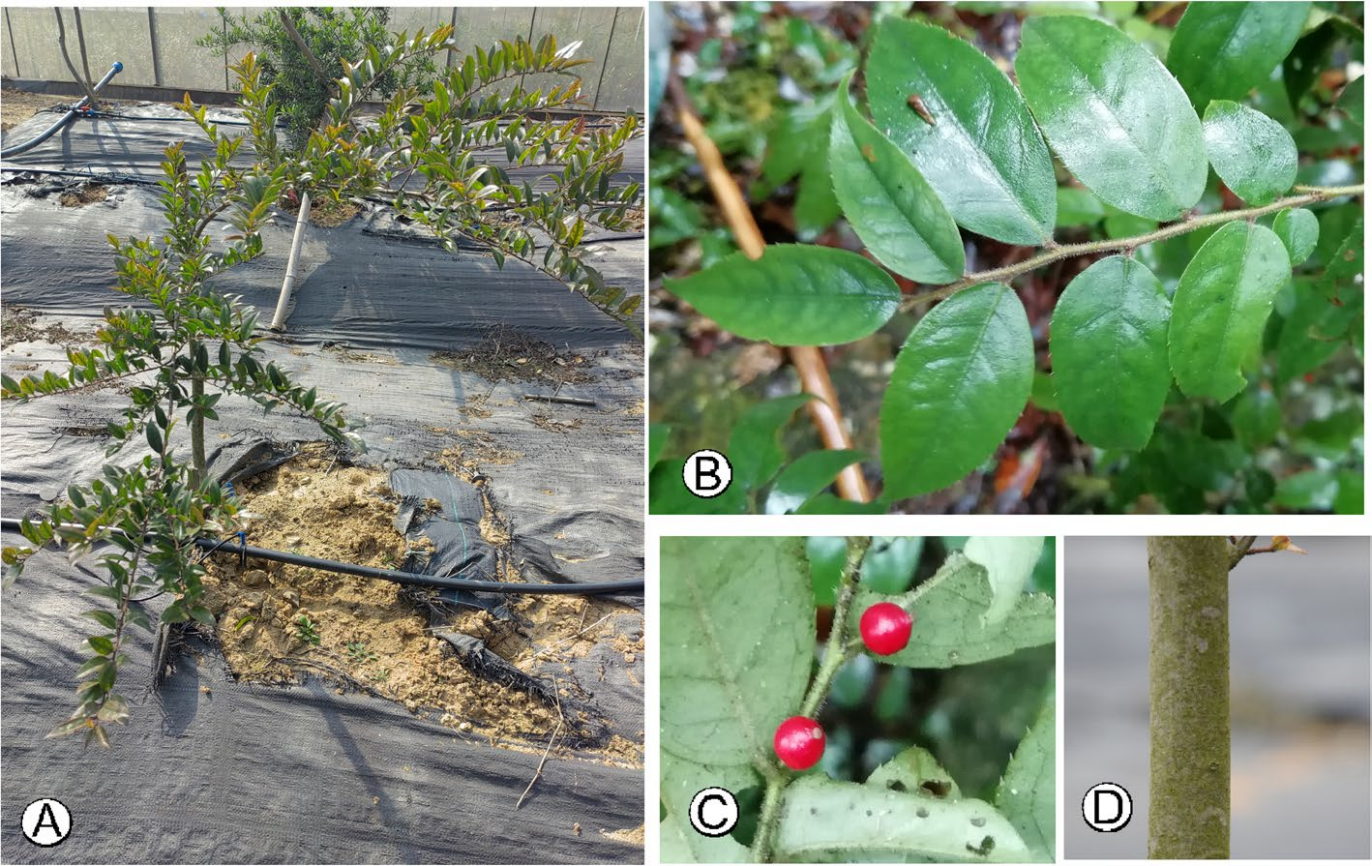
The morphological characteristics of *I. pubescens*. **A** The plant. **B** The leaves. **C** The branch with fruits. **D** The tree bark

Specimens of *I. pubescens* were obtained the national holly germplasm bank of China located at Jiangsu Academy of Forestry (Nanjing, Jiangsu, China). The materials came from populations under cultivation. The original source originated from a collection of seeds from natural population in the Wutong Mountains, Shenzhen, Guangdong, China (114°13′45″ E, 22°33′45″ N) in 2008. After stratification with temperatures below zero for 60–90 days, the seeds were germinated in seedling blocks and then were transplanted to the testing grounds of Jiangsu Academy of Forestry. For each sex of this plant, young leaf tissue from fifteen mature and well developed individuals, with different phenotypes, were selected in the the testing grounds for flow cytometry analysis. Fresh leaves from a single female individual (Fig. 7) were used to conduct genome survey sequencing. No specific permissions were required for the collection of specimens for this study which were neither privately owned nor protected and the field study did not involve endangered or protected species. We complied with the IUCN Policy Statement on Research Involving Species at Risk of Extinction and the Convention on the Trade in Endangered Species of Wild Fauna and Flora. After the formal identification of the plant material was carried out by Peng Zhou, voucher specimens were prepared and deposited at the herbarium of Nanjing Forestry University (NF, accession number NF202298).

Total genomic DNA was isolated using a DNA extraction kit (CWBIO, Shanghai, China). DNA integrity was monitored on 1% agarose gel electrophoresis and the purity was detected on NanoDrop 2000 (Thermo Fisher, USA). DNA concentration was measured using Qubit Flurometer (Thermo Fisher, USA).

### Genome size estimation by flow cytometry

The genome size was determined using the BD Influx™ cell sorter (BD, Piscataway, NJ, USA). Young leaves of the target species and a standard species were simultaneously processed by slightly modified procedures as described by Espejo et al. [45]. Approximately 50 mg of fresh leaf tissue from each plant were co-chopped in 1 mL pre-cooled Tris dissociation solution with a new razor blade. The mixture was filtered and collected in a centrifuge tube and centrifuged at 4 °C for 5 min at 1,000 rpm/min. The supernatant was discarded, and the pellet was stained with 500 μL propidium iodide (PI, 50 µg/mL) stain solution (containing 50 µg/mL RNase). Stained nuclei suspensions were incubated in the dark at 4 °C for 10 min and then filtered and loaded onto the flow cytometer for detection. A green argon laser with 488 nm wavelength was used to analyze over 5,000 nuclei in the sample. FACS sortware1.0.0.650 was used for capturing fluorescent signals and data analysis with the coefficient variation values (CV) of both peaks controlled in 5% [46]. *Oryza sativa* subsp. japonica cv. Nipponbare (1*C* = 389 Mb, GC = 43.6%) [47] with a known genome size served as internal reference standards. The genome size of each sample was calculated according to the following formula [46]: Sample genome size = [(sample G_0_/G_1_ peak mean)/(standard G_0_/G_1_ peak mean)] × standard genome size.

Variance analysis was carried out using Excel 2013 and SPSS 13.0 with convective detection parameters. A t test was performed to determine if there were differences in genome size between male and female, and *P* < 0.05 was considered statistically significant.

### Genome survey sequencing and quality control

The genomic paired-end libraries with 350-bp insertions were constructed on a DNBSEQ-T7 sequencing platform using *I. pubescens* following the guidance of the standard procedure (MGI Tech Co., Ltd) at the Compass Agritechnology Co., Ltd (Beijing, China). In order to ensure the quality of the analysis, we filtered the paired reads that would interfere with subsequent information, the paired reads with adapter contamination, the paired reads with uncertain nucleotides (N) constitute more than 10 percent of either read, and the paired reads when low quality nucleotides (base quality less than 5) constitute more than 50 percent of either read. Clean reads were obtained after filtering and correction of the sequence data.

### K-mer analysis

All of the clean data were used for k-mer analysis using Jellyfish software. Based on the results of k-mer frequency distributions (k-mer = 21), the characteristics of the genome, including genome size, heterozygosity and repeat rate, were estimated by using GenomeScope.

### *De novo* genome assembly and guanine plus cytosine (GC) content analysis

Genome sequence assembly was performed using the *de Bruijn* graph constructed based on the overlapping relationship reads from SOAPdenovo2 software. Contigs were realigned using all clean reads and scaffolds were constructed step by step using diversified insert size paired-ends. A k-mer size of 21 was set as the default assembly parameter. The resultant scaffolds longer than 300 bases in length were chosen. A window size of 10 kb was used for non-repetitive advancement in the sequence and calculation of the mean depth and GC content of every window to generate a GC depth plot.

### Identification and verification of microsatellite motifs

The MicroSatellite identification software (MISA) was used to identify microsatellite motifs in the scaffold. The settings implemented to detect the minimum numbers of SSRs for mono-, di-, tri-, tetra-, penta- and hexa-nucleotide repeats were as follows: number of mono-nucleotide repeats was less than 10, number of di-nucleotide repeats was less than 6, and numbers of remaining repeats were all less than 5, respectively.

## Supplementary Information

**Additional file 1.**

## Data Availability

Raw data and the genome assembly from this study were deposited in NCBI under the BioProject ID: PRJNA909012. The datasets supporting the conclusions of this article are included within the article.
