# Supplementary material for: A first insight into the genomic background of Ilex pubescens (Aquifoliaceae) by flow cytometry and genome survey sequencing
Source: BMC Genomics. 2023 May 19;24:270. doi: 10.1186/s12864-023-09359-5 (PMC10197237; doi:10.1186/s12864-023-09359-5)
Supplement: Supplementary file 1 — Additional file 1. [file 12864_2023_9359_MOESM1_ESM.pdf]

Table S1 The SSR types detected in the *Ilex pubescens* sequences.

SSR repeat type	Number	Proportion(%)
Mononucleotide	123333	62.47%
Dinucleotide	63069	31.95%
Tri-nucleotide	8971	4.54%
Tetra-nucleotide	1128	0.57%
Penta-nucleotide	388	0.20%
Hexa-nucleotide	540	0.27%
Dinucleotide		
AC/GT	14324	22.71%
AG/CT	34379	54.51%
AT/AT	14262	22.61%
CG/CG	104	0.16%
Tetranucleotide		
AAC/GTT	526	5.86%
AAG/CTT	2262	25.21%
AAT/ATT	2289	25.52%
ACC/GGT	2327	25.94%
ACG/CGT	90	1.00%
ACT/AGT	451	5.03%
AGC/GCT	131	1.46%
AGG/CCT	583	6.50%
ATG/CAT	243	2.71%
CCG/CGG	69	0.77%
